# Phylogenomic and evolutionary dynamics of inverted repeats across *Angelica* plastomes

**DOI:** 10.1186/s12870-020-02801-w

**Published:** 2021-01-07

**Authors:** Mengli Wang, Xin Wang, Jiahui Sun, Yiheng Wang, Yang Ge, Wenpan Dong, Qingjun Yuan, Luqi Huang

**Affiliations:** 1 State Key Laboratory Breeding Base of Dao-di Herbs, National Resource Center for Chinese Materia Medica, China Academy of Chinese Medical Sciences, Beijing, 100700 China; 2 Shenyang Medical College, Shenyang, 110034 China; 3 Laboratory of Systematic Evolution and Biogeography of Woody Plants, School of Ecology and Nature Conservation, Beijing Forestry University, Beijing, 100083 China

**Keywords:** *Angelica*, Plastome evolution, Phylogenomic, Inverted repeats

## Abstract

**Background:**

*Angelica* L. (family Apiaceae) is an economically important genus comprising ca. One hundred ten species. *Angelica* species are found on all continents of the Northern Hemisphere, and East Asia hosts the highest number of species. Morphological characters such as fruit anatomy, leaf morphology and subterranean structures of *Angelica* species show extreme diversity. Consequently, the taxonomic classification of *Angelica* species is complex and remains controversial, as the classifications proposed by previous studies based on morphological data and molecular data are highly discordant. In addition, the phylogenetic relationships of major clades in the *Angelica* group, particularly in the *Angelica* s. s. clade, remain unclear. Chloroplast (cp) genome sequences have been widely used in phylogenetic studies and for evaluating genetic diversity.

**Results:**

In this study, we sequenced and assembled 28 complete cp genomes from 22 species, two varieties and two cultivars of *Angelica*. Combined with 36 available cp genomes in GenBank from representative clades of the subfamily Apioideae, the characteristics and evolutionary patterns of *Angelica* cp genomes were studied, and the phylogenetic relationships of *Angelica* species were resolved. The *Angelica* cp genomes had the typical quadripartite structure including a pair of inverted repeats (IRs: 5836–34,706 bp) separated by a large single-copy region (LSC: 76,657–103,161 bp) and a small single-copy region (SSC: 17,433–21,794 bp). Extensive expansion and contraction of the IR region were observed among cp genomes of *Angelica* species, and the pattern of the diversification of cp genomes showed high consistency with the phylogenetic placement of *Angelica* species. Species of *Angelica* were grouped into two major clades, with most species grouped in the *Angelica* group and *A. omeiensis* and *A. sinensis* grouped in the *Sinodielsia* with *Ligusticum tenuissimum.*

**Conclusions:**

Our results further demonstrate the power of plastid phylogenomics in enhancing the phylogenetic reconstructions of complex genera and provide new insights into plastome evolution across *Angelica* L.

## Background

The herbaceous perennial genus *Angelica* L. (family Apiaceae) is a taxonomically complex and controversial group comprising approximately 110 species with extreme polymorphism in leaf morphology, fruit anatomy and subterranean structures [1–3]. Members of *Angelica* are distributed on all Northern Hemisphere, with the largest number of species (approximately 55) concentrated in East Asia [3–5]. Forty-five *Angelica* species are distributed in China with 32 of them endemic [3, 6]; some species are extremely rare in the field and are only known from limited specimens [7].

Some of these endemic *Angelica* species are of great economic value and have been used in traditional Chinese medicines for hundreds of years [3, 8]. Some species of *Angelica* are official *materia medica* recorded in Chinese Pharmacopoeia Committee of People’s Republic of China’s 2010, including *A. sinensis* (Chinese medicine name: *Danggui*), *A. biserrata* (*Duhuo*) and *A. dahurica* (*Baizhi*) [7]. Another 15 species of *Angelica* are also used as herbal medicinal materials in folk remedies (http://frps.eflora.cn).

Previous studies of *Angelica* systematics have focused on karyotaxonomical analyses [2, 9, 10], pollen morphology [11–13], petiole and fruit anatomy [14], and phytochemistry [15, 16]. Previous molecular phylogenetic analyses of *Angelica* have exclusively been based on phylogenetic analyses of DNA sequences, especially on the nuclear ribosomal (nr) DNA internal transcribed spacer (ITS) region, and relatively few Chinese representatives of *Angelica* have been included in analyses [6, 17–20]. Xue et al. (2007) used 44 ITS sequences from species of *Angelica* sensu stricto (s.s.) and allies from East Asia and proposed that *Angelica* was polyphyletic. Feng et al. (2009) suggested that *Angelica* s.s. was monophyletic after including *Coelopleurum*, *Czenaevia*, and *Ostericum koreanum* in analyses but excluding several other species previously recognized in *Angelica* s.l. Liao et al. (2013) reconstructed the phylogeny of *Angelica* s.l. and infrageneric relationships in *Angelica* s.s. with a more extensive sampling of *Angelica* species from East Asia (including 44 of its approximately 55 known species) and integrated analyses of nrDNA (ITS, ETS), cpDNA (*rps16* intron, *rps16-trnK* intergenic spacer, *rpl32-trnL* intergenic spacer, and *trnL-trnT* intergenic spacer), and morphological data. Their analysis suggested that many species of *Angelica* fell outside of *Angelica* s.s. and that four species of *Angelica* occurred outside of the *Angelica* group. However, the relationships of clades within the Selineae, particularly within the *Angelica* s.l. group, are still controversial and mostly unresolved.

Chloroplasts are key organelles for photosynthesis and other biochemical pathways in plants [21, 22]. The chloroplast (cp) genome is one of the three DNA genomes (with nuclear and mitochondrial genomes) in plants with a relatively conserved quadripartite circular structure ranging from 115 to 165 kb [23, 24]. Because of their relatively stable genome structure, the complete cp genome sequences have been widely accepted to provide a valuable and informative data source for understanding evolutionary biology and have become a powerful tool for resolving plant phylogenies [24–35].

In this study, we report 28 newly sequenced and complete cp genomes from the genus *Angelica* (22 species, two varieties and two cultivars) and investigate the structural diversity of cp genomes in *Angelica* by comparative chloroplast genome analyses. Furthermore, we test the power of complete cp genomes for resolving the phylogeny of the controversial and less well-resolved *Angelica* group by integrated analyses with another 36 published cp genomes available from NCBI GenBank from representative clades of the Apioideae (subfamily of Apiaceae).

## Results

### Characteristics of *Angelica* plastomes

The number of paired-end raw reads obtained by the Illumina HiSeq 4000 system ranged from 8,616,334 to 22,518,619 for the 28 *Angelica* samples. After mapping the paired-end reads of each *Angelica* taxon, 52,277 to 1,673,010 reads were extracted, yielding 59× to 1445× chloroplast genome coverage (Table 1). The inverted repeat (IR) junction regions in the assembled chloroplast genomes were further manually checked to avoid potential annotation errors. High-quality chloroplast genome sequences were thus achieved and facilitated for downstream analyses. The 28 *Angelica* chloroplast genome sequences were deposited in GenBank (accession numbers, MT921958-MT921985).

**Table 1:** Statistics of NGS sequencing of 28 *Angelica* samples

ID	Species	Raw reads no.	Mapped reads No.	Chloroplast genome coverage (x)
DG001	*A. morii*	11,122,925	522,264	546
DG002	*A. tianmuensis*	22,518,619	1,673,010	1445
DG003	*A. cartilaginomarginata* var. *foliata*	14,742,849	195,610	204
DG004	*A. biserrata*	19,320,868	129,002	136
DG005	*A. polymorpha*	13,445,469	258,026	290
DG006	*A. megaphylla*	9,985,839	133,105	141
DG007	*A. valida*	9,519,329	52,277	59
DG008	*A. decursiva*	14,307,154	419,462	430
DG009	*A. kangdingensis*	14,576,047	176,603	199
DG010	*A. apaensis*	11,056,070	964,507	1096
DG011	*A. maowenensis*	12,236,395	186,811	211
DG012	*A. pseudoselinum*	11,687,001	624,354	706
DG013	*A. laxifoliata*	11,817,482	216,804	245
DG014	*A. omeiensis*	18,910,215	1,243,559	1134
DG015	*A. tsinlingensis*	14,571,967	62,281	70
DG016	*A. dahurica* var. *formosana*	11,086,333	55,945	62
DG017	*A. dahurica* cv. ‘Qibaizhi’	10,575,208	500,975	569
DG018	*A. porphyrocaulis*	8,616,334	300,612	345
DG019	*A. dahurica* cv. ‘Qibaizhi’	9,073,284	231,034	266
DG020	*A. nitida*	9,991,134	70,752	80
DG023	*A. cartilaginomarginata*	14,135,856	145,751	163
DG025	*A. anomala*	10,508,302	277,618	313
DG026	*A. dahurica* cv. ‘Hangbaizhi’	12,266,363	296,498	336
DG027	*A. dahurica* cv. ‘Hangbaizhi’	9,171,688	990,934	1132
DG028	*A. sinensis*	9,212,560	929,902	947
DG029	*A. sinensis*	10,265,910	1,084,379	1188
HG021	*A. dahurica*	17,918,362	359,326	407
HG022	*A. gigas*	14,989,366	86,751	96

**Fig. 1:**
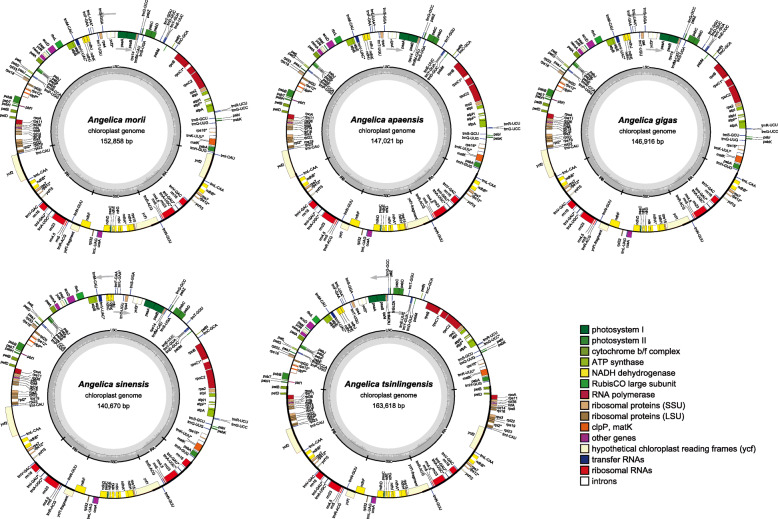
Gene map of five *Angelica* chloroplast genomes. The genes transcribed in the clockwise and counterclockwise directions are plotted inside and outside the circle, respectively. Different colors indicate genes belonging to different functional groups. The small single-copy (SSC) and large single-copy (LSC) regions are separated by the region of inverted repeats (IRa and IRb) indicated with the thick lines

The length of the complete chloroplast genome ranged from 140,670 bp (*A. sinensis*) to 163,618 bp (*A. tsinlingensis*) among the 33 cp genomes from 27 *Angelica* species (varieties or cultivars). All of the cp genomes possessed the typical quadripartite structure of angiosperms, including a pair of inverted repeat regions (IRs: 5836–34,706 bp) separated by a large single-copy region (LSC: 76,657–103,161 bp) and a small single-copy region (SSC: 17,433–21,794 bp) (Fig. 1; Table 2). The average GC content was 37.5%, which was virtually identical among the 33 complete *Angelica* cp genomes. The total number of genes ranged from 121 (*A. sinensis*) to 144 (*A. tsinlingensis*) in these 33 complete *Angelica* cp genomes. After removing the duplicated genes in IR regions, the 33 *Angelica* cp genomes harbored 113 to 114 different genes, including 80 protein-coding and 4 rRNA genes shared by all cp genomes (Table 1). While most cp genomes contained 29 tRNA genes, seven cp genomes contained one more tRNA gene (*trnG*-UCC or *trnG*-GCC) (Additional file 1: Table S1). The organization, gene order and GC content of cp genomes in *Angelica* were highly identical and similar to those of other higher plants (Fig. 1).

**Table 2:** Comparison of the chloroplast genome features of *Angelica* species

ID	Species	CP genome type	Genome size (bp)	LSC size (bp)	SSC size (bp)	IR size (bp)	Genome GC	Total genes	Protein coding genes	rRNA genes	tRNA genes	Total genes uniq
DG001	*A. morii*	I	152,858	86,914	17,524	24,210	37.5%	129	85	8	36	113
DG025	*A. anomala*	II	147,145	93,695	17,820	17,815	37.5%	127	84	8	35	113
DG010	*A. apaensis*	II	147,021	93,693	17,770	17,779	37.5%	127	84	8	35	113
DG004	*A. biserrata*	II	146,677	93,217	17,500	17,980	37.5%	128	84	8	36	114
DG023	*A. cartilaginomarginata*	II	146,583	94,185	17,806	17,296	37.5%	128	84	8	36	114
DG003	*A. cartilaginomarginata* var. *foliata*	II	147,017	93,622	17,777	17,809	37.5%	127	84	8	35	113
DG017	*A. dahurica* cv. ‘Qibaizhi’	II	146,815	93,547	17,690	17,789	37.5%	127	84	8	35	113
DG019	*A. dahurica* cv. ‘Qibaizhi’	II	146,811	93,547	17,630	17,817	37.5%	127	84	8	35	113
KT963037	*A. dahurica*	II	146,918	93,605	17,677	17,818	37.5%	128	85	8	35	113
DG026	*A. dahurica* cv. ‘Hangbaizhi’	II	146,810	93,546	17,630	17,817	37.5%	127	84	8	35	113
DG027	*A. dahurica* cv. ‘Hangbaizhi’	II	146,835	93,571	17,630	17,817	37.5%	127	84	8	35	113
HG021	*A. dahurica*	II	147,477	93,584	18,259	17,817	37.5%	128	84	8	36	114
DG016	*A. dahurica* var. *formosana*	II	147,097	93,322	17,625	18,075	37.5%	127	84	8	35	113
DG008	*A. decursiva*	II	146,158	92,655	17,537	17,983	37.6%	128	84	8	36	114
DG009	*A. kangdingensis*	II	146,529	93,853	17,530	17,573	37.5%	128	84	8	36	114
DG013	*A. laxifoliata*	II	146,682	93,964	17,532	17,593	37.5%	127	84	8	35	113
DG011	*A. maowenensis*	II	146,882	94,105	17,433	17,672	37.4%	127	84	8	35	113
DG006	*A. megaphylla*	II	146,724	92,350	17,672	18,351	37.5%	127	84	8	35	113
DG020	*A. nitida*	II	146,789	93,081	17,506	18,101	37.4%	127	84	8	35	113
MF594405	*A. nitida*	II	146,512	103,161	21,794	5836	37.5%	127	84	8	35	113
DG014	*A. omeiensis*	II	147,814	93,787	17,635	18,196	37.6%	128	85	8	35	113
DG005	*A. polymorpha*	II	146,982	93,442	17,754	17,893	37.6%	127	84	8	35	113
DG018	*A. porphyrocaulis*	II	146,859	93,543	17,682	17,817	37.5%	128	84	8	36	114
DG012	*A. pseudoselinum*	II	148,942	91,035	17,527	20,190	37.5%	128	85	8	35	113
DG002	*A. tianmuensis*	II	147,308	93,239	17,637	18,216	37.5%	127	84	8	35	113
DG007	*A. valida*	II	146,833	94,103	17,530	17,600	37.5%	127	84	8	35	113
KT963036	*A. acutiloba*	II	147,074	93,368	17,574	18,066	37.5%	128	85	8	35	113
KX352468	*A. acutiloba*	II	147,074	93,368	17,574	18,066	37.5%	128	85	8	35	113
HG022	*A. gigas*	III	146,916	93,163	17,579	18,087	37.6%	127	84	8	35	113
KT963038	*A. gigas*	III	146,916	93,119	17,583	18,107	37.6%	128	85	8	35	113
DG028	*A. sinensis*	IV	140,694	101,695	17,591	10,704	37.4%	121	80	8	33	113
DG029	*A. sinensis*	IV	140,670	101,680	17,578	10,706	37.5%	121	80	8	33	113
DG015	*A. tsinlingensis*	V	163,618	76,657	17,549	34,706	37.5%	144	99	8	37	114

**Fig. 2:**
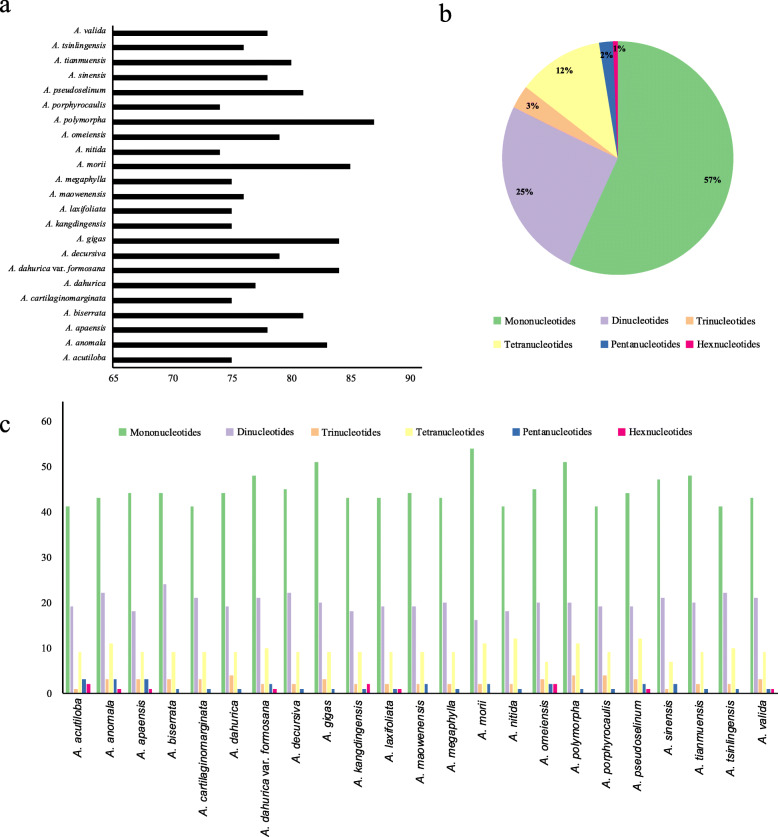
Comparison of simple sequence repeats among 33 *Angelica* chloroplast genomes. **a** Number of SSRs detected in 33 *Angelica* chloroplast genomes; **b** Frequencies of identified SSRs in different repeat types; **c** Number of SSRs in different repeat types in 33 *Angelica* chloroplast genomes

The number of simple sequence repeats (SSRs) ranged from 68 (*A. nitida*) to 87 (*A. polymorpha*) among the 33 *Angelica* cp genomes (Fig. 2a). Most of the SSRs were mono-nucleotide repeats (58%), while di-nucleotide, tri-nucleotide, tetra-nucleotide, penta-nucleotide and hex-nucleotide SSRs made up 24, 4, 11, 2 and 1% of all SSRs, respectively (Fig. 2b). The mono-nucleotide repeat number with the highest variability, ranged from 38 (*A. nitida*) to 54 (*A. morii*), while the number of other repeat types did not significantly differ among the 33 *Angelica* cp genomes (Additional file 2: Table S2, Fig. 2c).

### Expansion and contraction of the IR region

**Fig. 3:**
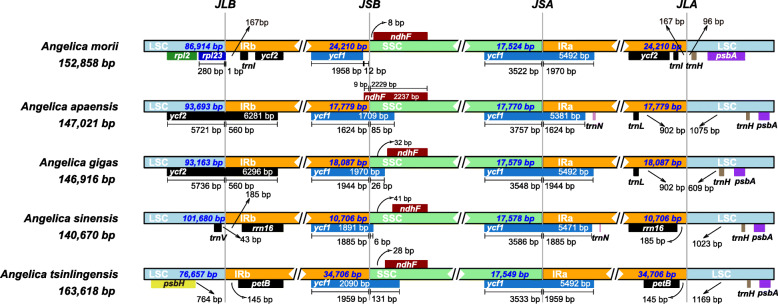
Comparison of the borders of LSC, SSC, and IR regions of chloroplast genomes in six *Angelica* species

**Fig. 4:**
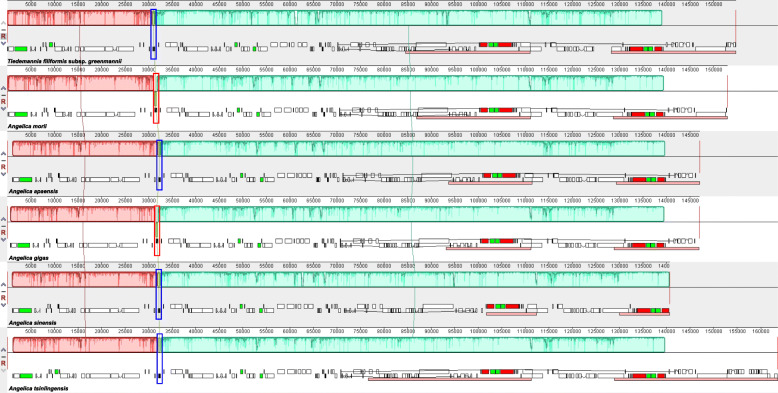
MAUVE alignment of chloroplast genomes in six *Angelica* species. The chloroplast genome of *Tiedemannia filiformis* subsp. *greenmannii* is shown at the top as the reference genome. Within each of the alignments, local collinear blocks are represented by blocks of the same color connected by lines

Although cp genomes are highly conserved in genomic structure and size, the change in the size of the IR/SC junction caused by the expansion and contraction of the IR/SC boundary regions has been considered a primary mechanism for creating length variation in cp genomes of higher plants [26, 36–38]. Extensive expansion and contraction of the IR region were observed among the 33 *Angelica* cp genomes examined in this study and could be classified into five different types based on the characteristics in the IR/SC junction region and with/without inversion. The IR region of *A. morii* expanded and contained a duplicate copy of the *ycf2* gene (Type I); in most (25/33) *Angelica* cp genomes, the junction site of IR/SSC was located in the *ycf1* gene, and the junction site of IR/LSC was located between genes of *trnL* and *trnH* (Type II) (Fig. 3). An inversion of approximately 490 bp in the *trnY-trnD-trnE* gene was observed in the cp genome of *A. gigas* (Type III) and in *A. moii* (Fig. 4). Significant contraction of the IR region was detected in *A. sinensis* (10,706 bp) and ended with the *rrn16* gene in the IR region (Type IV); the largest expansion of the IR region was observed in *A. tsinlingensis* and ended with the *petB* gene in the IR region (Type V) (Fig. 3).

### Phylogenetic analysis

**Fig. 5:**
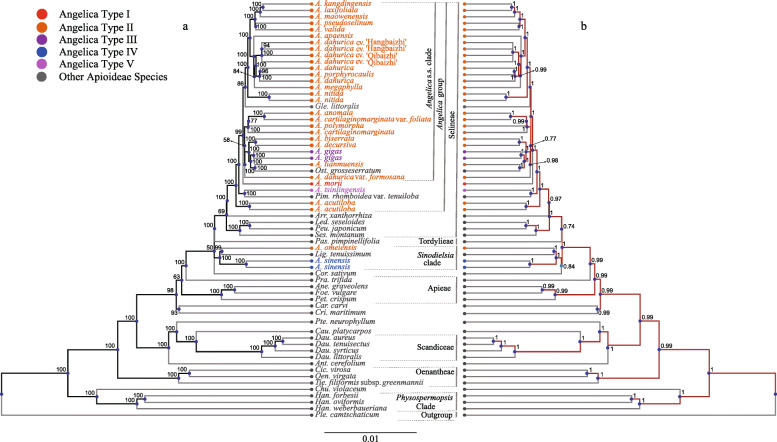
Phylogenetic trees derived from analyses of chloroplast genomes from 33 *Angelica* and 31 other representative Apioideae species. **a** Majority-rule consensus tree derived from maximum likelihood (ML) analysis. Numbers at each node are bootstrap values calculated from 2000 replicates. **b** Majority-rule consensus tree derived from Bayesian (BI) analysis. Numbers at each node are posterior probability estimates from 2 × 5,000,000 MCMC generations with sampling every 1000 generations. Different colors are used to indicate different types of chloroplast genomes based on characteristics of the LSC, SSC, and IR boundary regions

The ML and Bayesian trees yielded highly similar topologies. Members of *Angelica* fell primarily into two major lineages: (1) the *Angelica* group occurring in tribe Selineae (BS = 100, PP = 1), and (2) the *Sinodielsia* clade (BS = 100, PP = 1) (Fig. 5). The names of major clades determined by previous studies are followed [1, 39–41]. The *Angelica* group made up most of the *Angelica* accessions (30/33), and 26 *Angelica* accessions formed the well-supported *Angelica* s.s. clade (BS = 99, PP = 1) which also included *Glehnia littoralis* and *Ostericum grosseserratum* (Fig. 5). Within the *Angelica* s.s. clade, four major lineages were recovered (*A. kangdingensis* to *A. valida*, *A. apaensis* to *A. megaphylla*, *A. anomala* to *A. cartilaginomarginata*, and *A. biserrata* to *Ost. grosseserratum*). The support value of the placement of clade *A. anomala* to *A. dahurica* var. *formosana* was relatively low (BS = 58, PP = 0.77). The littoral *Angelica* species *A. morii*, which inhabits the East Asian littoral regions or islands, and *A. tsinlingensis*, which is clearly different from members of the *Angelica* s.s. clade by its thin-winged dorsal ribs and triple vittae in the furrow [1], were placed outside of the *Angelica* s.s. clade based on the molecular findings (Fig. 5). *A. acutiloba* is also isolated from the *Angelica* s.s. clade and occupies an early diverging branch of the *Angelica* group (Fig. 5). The *Sinodielsia* clade consisted of *A. omeiensis*, *A. sinensis* and *Ligusticum tenuissimum.* Most clades in the *Angelica* group received high BS/PP support with the exception of the clade that included *A. anomala* to *A. dahurica* var. *formosana* (BS = 58, PP = 0.77) (Fig. 5). Most accessions of *A. dahurica* (*A. dahurica*, *A. dahurica* cv. *hangbaizhi* and *A. dahurica* cv. *xingan*) were placed in a well-supported clade that also included *A. porphyrocaulis*, with the exception of *A. dahurica* var. *formosana*, which was placed in a relatively distant clade that included *A. anomala* to *A. tianmuensis* and *Ostericum grosseserratum* (Fig. 5). Clades of non-*Angelica* species were generally consistent with those inferred by previous studies [1, 6, 40, 42, 43].

## Discussion

### Expansion and contraction of the IR region in *Angelica* Plastomes

In this study, we sequenced 28 chloroplast genomes of *Angelica* using the Illumina HiSeq-4000 platform and performed comparative analyses of these genomes with five other published chloroplast genomes of the same genus available from GenBank. The chloroplast genomes of *Angelica* had a typical quadripartite structure of higher plants, were conserved in gene order and content and consisted of 113 to 114 different genes. The cp genomes among *Angelica* species were similar in GC content, but the GC contents in LSC and SSC regions were significantly lower than those in the IR region because of the inclusion of eight rRNA genes with high GC contents in the IR region. The IR region is considered the most conserved region of the chloroplast genome [44].

The primary causes of differences in the lengths of chloroplast genomes are considered to be the expansion and contraction in IR, LSC, and SSC regions, which are relative common during evolution [45].

The lengths of cp genomes varied between 140,670 base pairs (*A. sinensis*) to 163,618 base pairs (*A. tsinlingensis*). Shrinkage, expansion, or loss of the IR region has been proposed to be one of the main reasons explaining the change in the size of cp genomes [46]. Large-scale expansion and contraction of the IR region were reported in Apiaceae; indeed the frequency and large size of JLB shifts documented in Apioideae cp genomes are unprecedented among the angiosperms [47]. In our study, extensive expansion and contraction of the IR region were detected in the *Angelica* species, with five types of changes in the IR and boundary between IR and SSC or LSC of the chloroplast genomes discovered (Fig. 3). Most *Angelica* species (25/33) had Type II cp genomes with the junction site of IR/SSC located in the *ycf1* gene and the junction site of IR/LSC located between the genes *trnL* and *trnH* and were significantly clustered in the *Angelica* s.s. group, with the exception of *A. omeiensis*, which was grouped in the *Sinodielsia* clade*.* The expansion of the IR region results in the inclusion of extra genes in this region; for example, expansion of this region in the littoral species *A. morii* resulted in the inclusion of a duplicated copy of the *ycf2* gene (Type I). The largest expansion of the IR region was observed in the cp genome of *A. tsinlingensis* (34,706 bp) and ended with the *petB* gene in the IR region. A significant contraction of the IR region was observed in *A. sinensis* (10,704 bp). The patterns of variation observed were generally consistent with the groups of *Angelica* species recovered in the phylogenetic analyses, reflecting the high diversification of species and cp genomes in this controversial genus (Fig. 5).

### Phylogenetics of the genus *Angelica*

With the use of the whole cp genome sequence from 33 *Angelica* species and another 31 representative species of Apioideae, a highly consistent topology was recovered by ML and Bayesian analyses (Fig. 5). The allocation of the main clades of Apioideae (e.g., Oenantheae, Scandiceae, Apieae, Tordylieae, and Selineae) were consistent with those inferred by previous studies [18, 40, 42, 43]. Species of *Angelica* were not grouped in a monophyletic clade but distributed in four clades, with most *Angelica* species grouped in a well-supported clade (the *Angelica* group), supporting the phylogenetic topologies of previous studies [1, 41]. This group also consisted of species from the genus *Glehnia* (*Gle. littoralis), Ostericum (Ost. grosseserratum)* and *Pimpinella (Pim. rhomboidei* var. *tenuiloba).*

The *Angelica* s.s. clade in Liao et al. (2013) primarily contained East Asian *Angelica* species and species from *Ostericum* (*Ost. Koreanum, Ost. huangdongensis)* and *Czemaevia (Cze. Laevigata* var. *larvigata)* but excluded species from *Glehnia (Gle. littoralis* var*. littoralis, Gle.* var*. leiocarpa)*. Based on whole cp genome data, *Gle. littoralis* was grouped within the *Angelica* s.s. clade with relatively high support (BS = 86, PP = 1). *A. anomala* was previously grouped with *Ostericum grosseserratum* and species from *Peucedanum* within the *Acronema* clade [6, 43] based on nrITS sequences but was then placed into the *Angelica* s.s clade when both nrITS, nrETS, cpDNA and morphological characters were used [1]*.* In the study, *A. anomala* was grouped with *A. cartilaginomarginata, A. cartilaginomarginata* var. *foliata,* and *A. polymorpha* in a clade within the *Angelica* s.s. clade. The allocation of *A. morii* and *A. tsinglingensis* within the *Angelica* group but outside the *Angelica* s.s. clade was also consistent with previous studies and was supported by studies of morphological characters (e.g., dorsal ribs and triple vittae in each furrow) [1]. Because of its unusual fruit characteristics, the taxonomic position of *A. acutiloba* has been controversial for many years. *A. acutiloba* was previously placed within the *Angelica* s.s. clade based on nrDNA ITS sequences [6] and was then placed outside the *Angelica* s.s. clade with data from nrDNA, cpDNA, and morphological characters [1]. In our study, *A. acutiloba* was also isolated from the *Angelica* s.s. clade and occupies an early diverging branch of the *Angelica* group. *A. ameiensis* was previously grouped with *A. apaensis* and *A. nitida* in a clade within the *Angelica* s.s. clade based on nrDNA ITS and cpDNA sequences [1, 6] but was grouped with *A. sinensis* and *Ligusticum tenuissimum* in the *Sinodielsia* clade with high support (BS = 100, PP = 1) by whole cp genome data from this study.

This study reports the results of a comparative analysis of 33 *Angelica* cp genomes and found extensive expansion and contraction in the IR region among species of *Angelica*. The changes in cp genomes can be classified into five types that are consistent with the general phylogenetic placement of these *Angelica* species. The relationships of *Angelica* species examined here are were clear, and the lineages within the *Angelica* group were classified with a better resolution compared with previous studies. We suggest that the results of our study facilitate our understanding of the evolutionary history of *Angelica* species; nevertheless, more extensive cp genome sampling (e.g., *A. roseana, A. ampla, A. hirsutiflora,* and *A. oncosepala*) may be necessary to further characterize the relationships between *Angelica* species. These findings also provide an informative and valuable genetic source for *Angelica* germplasm resources to aid species identification and future taxonomic reconstructions of *Angelica*.

## Conclusions

Our analyses not only reveal extensive expansion and contraction of the IR region among cp genomes of *Angelica sp*ecies, but also show the power of plastome for resolving relationships in currently less-resolved and controversial groups. The variation patterns of IR region can be classified into five different types and are generally consistent with the groups of *Angelica* species in phylogenetic analyses. The relationships of *Angelica* species investigated here are mainly clearly classified and the lineages within the *Angelica* group are classified with a better resolution than previous studies, which we believe will facilitate the understanding of the evolutionary history of *Angelica* species, yet more extensive cp genome sampling may be necessary to further illustrate the relationships of species in *Angelica*.

## Methods

### Taxon sampling

We sampled 24 species (including two varieties and two cultivars) of *Angelica* located in 14 provinces, representing approximately 85% species and covering most of the distribution of *Angelica* in China (http://freps.eflora.cn/). Details of sampling information of the 28 samples collected in this study were shown in Supporting information Additional file 3: Table S3. All the samples were identified by Nian-He Wang (Institute of Botany, Jiangsu Province and Chinese Academy of Sciences) based on the morphological characters and the species were preserved in the herbarium of National Resource Center for Chinese Materia Medica, China Academy of Chinese Medical Sciences. Permission was not necessary for collecting these samples, which have not been included in the list of national key protected plants. The fresh leaves from each accession were immediately dried with silica gel for further DNA extraction.

### Plant material and DNA extraction

The Plant Genomic DNA Kit (DP305) from Tiangen Biotech (Beijing) Co., Ltd., China was used to extract total genomic DNA from each sample. Both a NanoDrop spectrophotometer (ND-1000; Thermo Fisher Scientific, USA) and a Qubit 2.0 fluorometer (Invitrogen, Life Technologies) were used to assess the quality and quantity of DNA.

### Illumina sequencing

A Covaris S2 was used to fragment total genomic DNA (30–150 ng) to a mean fragment size of 550 bp. The TruSeq DNA Nano Library Prep kit (Illumina) was used for DNA libraries preparation per the manufacturer’s instructions. Libraries were quantified using a KAPA Illumina Library Quantification Kit (KAPA Biosystems) by quantitative polymerase chain reaction, and the pooled libraries were sequenced (2 × 150 bp) using the Illumina HiSeq 4000 platform (Illumina, San Diego, CA).

### Chloroplast genome assembly and annotation

The raw sequencing reads were qualitatively assessed and assembled using the GetOrganelle version 1.6.4 [48] with default settings. Manual revision was performed to confirm the ambiguous nucleotides or gaps and the four junction regions between the IRs and SSC/LSC in the chloroplast genome sequences. The annotation of chloroplast genomes was performed using the GeSeq version 1.79 [49]. The annotation results were further manually checked using Geneious version 8.0.2 (http://www.Geneious.com) to avoid potential annotation errors. The gene maps of chloroplast genomes were plotted with OGDRAW version 1.3.1 [50].

### Simple sequence repeat analysis

The simple sequence repeat (SSRs or microsatellites) loci in the cp genomes were searched using the Perl script MISA version 2.0 [51]. The minimum numbers (thresholds) of the SSRs for mono-, di-, tri-, tetra-m penta-, and hexa-nucleotides, were 10, 5, 4, 3, 3, and 3 respectively. Manual verifications of the repeats were performed with abundant results removed.

### Comparative analysis of cp genomes

The statistics of genome size, GC content, LSC/SSC/IR size and number of genes were summarized using in-house python scripts. Comparative analysis of cp genome structure and gene content was performed using Mauve version 2015-02-13 [52] to locate potential rearrangements (e.g., inversion) and changes in gene order using the cp genome of *Tiedemannia filiformis* subsp. *Greenmannii* (GenBank accession: HM596071). The junction sites of LSC-IRa/b and SSC-IRa/b were checked by visualization using IRscope [53].

### Phylogenetic analysis

Phylogenetic analysis was conducted using all 33 *Angelica* cp genomes together with 31 species from major lineages of the subfamily Apoideae (Additional file 4: Table S4). The best-fit substitution models were selected using the PartitionFinder 2 version 2.1.1 [54] for Maximum likelihood (ML) and Bayesian inference (BI). The ML analyses were performed using RAxML-NG version 0.9.0 [55] with the general time-reversible (GTR) + G model, and node support was assessed with 2000 bootstrap replicates. The BI analyses were performed with MrBayes version 3.2.7a [56]. Two chains of 5,000,000 generations were performed for the Markov chain Monte Carlo (MCMC) analysis with trees sampled every 1000 generations. The first 25% of the sampled trees were discarded as burn-in and the remaining trees were used to build a 50% majority-rule consensus tree. Stationarity was considered achieved when the average standard deviation of split frequencies remained below 0.001.

## Supplementary Information

**Additional file 1: Table S1. **Gene content in 64 Apioideae chloroplast genomes.

**Additional file 2: Table S2.** Number of SSR loci detected in 64 Apoideae samples.

**Additional file 3: Table S3.** Sampling information of 24 species (including two varietas and two cultivars) of Angelica.

**Additional file 4: Table S4.** Comparison of the chloroplast genome features of 31 Apoideae species.

## Data Availability

All sequences used in this study are available from the National Center for Biotechnology Information (NCBI) (see Additional file 3: Table S3 and Additional file 4: Table S4). All raw reads are available in the short sequence archive under accession no. PRJNA684804.

## Abbreviations

BI: Bayesian Inference
GTR: General time reversible
IR: Inverted repeat
ITS: Internal transcribed spacer of ribosomal DNA
LSC: Large single copy
ML: Maximum Likelihood
rRNA: Ribosomal RNA
SSC: Small single copy
tRNA: Transfer RNA
